# The *Journal of Comorbidity* affiliates with the Society for Academic Primary Care

**DOI:** 10.15256/joc.2016.6.88

**Published:** 2016-07-04

**Authors:** Susan M. Smith, Joanne Reeve, Jane Gunn, Nathan R. Hill, Martin Fortin, Catherine A. O’Donnell, Marjan van den Akker, Joanne Protheroe, Stewart W. Mercer

**Affiliations:** ^1^HRB Centre for Primary Care Research, Department of General Practice, RCSI Medical School, Dublin, Ireland; ^2^Warwick Primary Care, Warwick Medical School, The University of Warwick, Coventry, UK; ^3^Primary Care Research Unit, Department of General Practice, University of Melbourne, Melbourne, Victoria, Australia; ^4^Nuffield Department of Primary Care Health Sciences, Oxford University, Oxford, UK; ^5^Department of Family Medicine and Emergency Medicine, Université de Sherbrooke, Sherbrooke, Quebec, Canada; and Centre intégré universitaire de santé et de services sociaux du Saguenay-Lac-St-Jean, Quebec, Canada; ^6^General Practice and Primary Care, Institute of Health and Wellbeing, University of Glasgow, Glasgow, UK; ^7^Department of Family Medicine, School of Public Health and Primary Care (CAPHRI), Maastricht University, Maastricht, The Netherlands; and Department of General Practice, KU Leuven, Leuven, Belgium; ^8^Keele University Institute for Primary Care and Health Sciences, Keele, Staffordshire, UK; ^*^Co-Editors-in-Chief of the *Journal of Comorbidity*; ^†^Officers of the Society for Academic Primary Care

**Keywords:** comorbidity, multimorbidity, multiple chronic conditions, open access, primary care

The *Journal of Comorbidity* and the Society for Academic Primary Care (SAPC) are pleased to announce an exciting new partnership aimed at strengthening collaborations and enhancing opportunities among primary care professionals with an interest in comorbidity and multimorbidity. The *Journal of Comorbidity* and SAPC share a mutual goal to improve the management and care of patients by making clinical and research information and perspectives available to a global network of healthcare professionals. This new partnership will be an invaluable contribution to expanding the research platform for discussions and the scholarly exchange of knowledge, ideas, and research on comorbidity and multimorbidity.

## Society for Academic Primary Care

SAPC provides a clear voice and a strong presence for academic primary care in the complex and ever-changing primary care environment. The Society offers a point of reference and contact for those seeking academic solutions to the problems they face in the provision and study of primary care, and the advancement of academic primary care.

Academic general practice was present from the inception of the UK National Health Service (NHS), and the Society has been at the heart of that community for over four decades. The first independent academic department was established at the University of Edinburgh in 1956. Other departments emerged, and in 1972, held their first scientific meeting. In 1974, a new society (the Association of University Teachers of General Practice, and later the Association of University Departments of General Practice) was formed. A growing recognition of the role and contribution of the wider academic primary care community led to the group changing its name to the Society for Academic Primary Care in 2000 [1].

To facilitate this endeavour, the SAPC holds annual and regional conferences that bring together hundreds of researchers and educators from the primary care community to showcase their latest work on all aspects and issues relating to primary care. The annual conference not only attracts delegates from the UK but also includes professionals from across the globe who share their experiences and research findings, attend workshops and special interest sessions, and network with like-minded primary care professionals. More recently, the annual SAPC conference has become an important avenue for discussing and disseminating research on multimorbidity.

The partnership with the *Journal of Comorbidity* comes at a historic time, as multimorbidity is now the norm, not the exception. Understandably, interest in and awareness of multimorbidity are growing, but much more knowledge, research, and guidance on the care, treatment, and understanding of multimorbidity is desperately needed. SAPC has had a long-standing interest in, and is committed to, building affiliations between the academic community and publishers with primary care interests. This work contributes to the three pillars of SAPC activity to:

- Raise the profile of academic primary care and SAPC to a wider audience
- Support workforce career development, including increased access to various publishing opportunities
- Enhance impact through collaboration.

These pillars closely align with the *Journal of Comorbidity’s* mission, which is to provide a high-quality resource to fulfil the following objectives:

- Highlight comorbidity/multimorbidity as a fundamental component of overall patient care
- Enhance the understanding of comorbidity/multimorbidity
- Identify and fill gaps in the evidence on comorbidity/multimorbidity
- Provide comprehensive guidance on preventing and managing comorbidity/multimorbidity
- Promote the collaboration of multidisciplinary healthcare providers
- Facilitate daily decisions in clinical practice.

SAPC is delighted to be working in partnership with the *Journal of Comorbidity*. It is just the beginning of SAPC’s renewed efforts to increase publishing collaborations and to provide its members with a broader platform of engagement with a key partner who shares our goal to provide useful and accessible health information. We look forward to working with the journal’s Editors in widening the reach, impact, and engagement with key researchers in the field of multimorbidity.

## Journal of Comorbidity

The *Journal of Comorbidity* is an international, open-access, peer-reviewed journal that aims to optimize the management of patients with comorbidity and multimorbidity. The journal features original clinical and experimental research articles, guidelines, policies, editorials, commentaries, protocols, and critical review articles, as well as proceedings of congresses. The Editorial Board also welcomes ideas and suggestions for special issues dedicated to unique themes.

The affiliation between the *Journal of Comorbidity* and SAPC is an important move in encouraging SAPC members to consider the *Journal of Comorbidity* as the premier journal for publishing their work related to comorbidity and multimorbidity. As a benefit of this partnership, SAPC members will receive a discounted rate to publish in the journal.

The Editorial Board is very excited to partner with SAPC and look forward to working with them over the coming years, and to the active participation of its members. We are confident that the journal, in partnership with SAPC, will continue to grow and ultimately increase its impact in this important research field.

## References

[1] Howie J, Whitfield M (2011). Academic general practice in the UK medical schools, 1948–2000: a short history.

